# Intestinal invagination caused by circumferential contraction with longitudinal relaxation of the wall

**DOI:** 10.1007/s10237-025-02010-0

**Published:** 2025-09-03

**Authors:** Hitomi Okino, Hironori Takeda, Shunichi Ishida, Yohsuke Imai

**Affiliations:** 1 Graduate School of Engineering, Kobe University, 1-1 Rokkodai, Nada, Kobe, 657-8501 Japan; 2 Graduate School of Engineering, Kyoto University, 53 Shogoin-Kawahara-cho, Sakyo, Kyoto, 606-8507 Japan; 3 Institute for Life and Medical Sciences, Kyoto University, 53 Shogoin-Kawahara-cho, Sakyo, Kyoto, 606-8507 Japan

**Keywords:** Intussusception, Muscle contraction and relaxation, Buckling deformation, Continuum mechanics

## Abstract

Pediatric intussusception is frequently observed in the ileocecal region, where the terminal ileum invaginates into the colon. Previous studies have indicated an association between pediatric intussusception and inflammation as well as intestinal motility. However, the underlying mechanisms remain unclear, particularly with regard to the mechanics. We hypothesized that invagination occurs when longitudinal and circular smooth muscles are not coordinated during peristalsis. To test the hypothesis from a mechanical perspective, we developed a computational model of the terminal ileum, where the terminal ileum is modeled as a hyperelastic tube. We showed that circumferential contraction with longitudinal relaxation of the hyperelastic tube wall caused invagination in the contracting region of the tube. We also found that invagination occurred when a square-shaped contracting region emerged in the hyperelastic tube. These results indicate that uncoordinated motion of the circular and longitudinal muscles can lead to invagination of the intestinal wall. In addition, the configuration of peristalsis may serve as an indicator of the risk of pediatric intussusception.

**Supplementary Information:**

The online version contains supplementary material available at 10.1007/s10237-025-02010-0.

## Introduction

Intussusception is the invagination of one bowel segment into an adjacent segment. It is more prevalent in children than in adults, accounting for approximately $95\%$ of all the cases (Marinis et al. 2009). Pediatric intussusception is an acute abdominal emergency. Early diagnosis and treatment are required because delayed treatment can lead to serious complications such as necrosis of the intestinal wall and even death (Marsicovetere et al. 2016).

Disorders associated with intestinal motility are considered major causes of intussusception. Abnormal wall motions occur in the presence of lesions (Marinis et al. 2009; Marsicovetere et al. 2016), and inflammation caused by infectious diseases. Several studies have suggested that infectious diseases are associated with pediatric intussusception (Handa et al. 2021; Bines et al. 2006; Nylund et al. 2010). Reymond (1972) proposed a possible mechanism of intussusception: intussusception can be induced by the contraction of an inhomogeneity region, with an area that is unable to deform due to the presence of a lesion or a polyp. However, the detailed processes and conditions of intussusception remain unclear.

Here, we focus on the coordination of circumferential smooth muscles with longitudinal smooth muscles. Intestinal wall motions are generated by the contraction and relaxation of smooth muscles in the wall, where the circular smooth muscle contracts and relaxes in the circumferential direction, and the longitudinal smooth muscle contracts and relaxes in the longitudinal direction. These two directional muscles work in coordination. For example, in peristalsis, contraction of the circular muscle begins shortly after the start of the contraction of the longitudinal muscle (Korsapati et al. 2008; Nicosia et al. 2001; Pouderoux et al. 1997; Hur et al. 2021), but the contractions of the two muscles peak at almost simultaneously (Korsapati et al. 2008). We hypothesize that invagination occurs when the two smooth muscles are not coordinated, specifically when the circular smooth muscle contracts while the longitudinal smooth muscle relaxes.

The invagination of the intestinal wall may be modeled as a continuum mechanics problem: the buckling deformation of a hyperelastic tube. The buckling of thin- and thick-walled tubes under compression has been widely studied (Zhu et al. 2013; Yu et al. 2024; Hunt et al. 2003). However, the effect of presence of contracting regions on buckling deformation is not well understood. Therefore, the purpose of this study is to clarify the deformation of a hyperelastic tube by circumferential contraction with longitudinal relaxation. We show that the circumferential contraction with longitudinal relaxation causes the invagination of the tube. We also show that the tube invagination occurs when the tube forms a square-shaped contracting region. We discuss the relevance of these results to actual intussusception.

## Method

### Problem statement

**Fig. 1:**
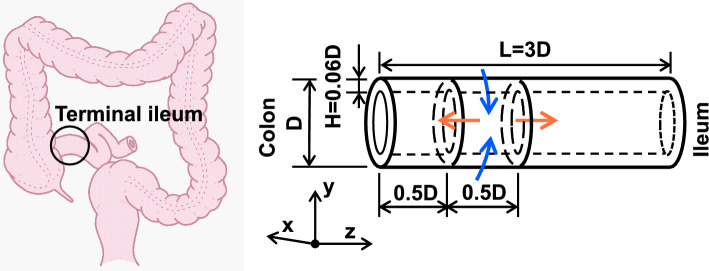
The terminal ileum is modeled as a hyperelastic tube with outer diameter *D*, length *L*, and wall thickness *H*. Circumferential contraction and longitudinal relaxation is given to the tube ($0.5D \le z \le 1.0D$)

Most pediatric intussusceptions are observed in the ileocecal region, where the ileal segment invaginates into the colon (Ein and Stephens 1971). The intestinal wall is composed of four layers: mucosa, submucosa, muscularis propria, and serosa, but for simplicity, we assume that these layers all have the same mechanical properties. We also assume that mucosal folds are small in the terminal ileum. Based on these assumptions, the terminal ileum of a child’s intestine is modeled as a three-dimensional, circular, single-layer hyperelastic tube with outer diameter *D*, length *L*, and wall thickness *H* as illustrated in Fig. 1. The length and thickness are defined as $L =$ 3*D* and $H =$ 0.06*D*, respectively, based on the actual size of a human child’s intestine in the ileocecal region (Haber and Stern 2000; Haworth et al. 1967). The terminal ileum is bound by the most distal bend of the ileum and the ileocecal junction, and the tube is assumed to be fixed at both ends in the colon and ileum sides.

Pediatric intussusception often occurs before the age of 1 year, when the intestinal contents have low viscosity. The elastic force induced by muscle contraction is significantly greater than the viscous force induced by the intestinal contents, and the effect of the intestinal contents can be omitted.

In this study, assuming that the circular and longitudinal muscle behaviors are not coordinated, circumferential contraction and longitudinal relaxation were considered to occur simultaneously in the wall. Contraction and relaxation of the intestinal wall result from a change in the length of the smooth muscles composing the intestinal wall. The contraction and relaxation of the wall are thus modeled by spontaneous plastic deformation, which changes the reference configuration.

### Spontaneous plastic deformation

For spontaneous plastic deformation, we use the finite growth theory proposed by Rodriguez et al. (1994). The deformation gradient tensor $\varvec{F}$ is decomposed into spontaneous plastic deformation $\varvec{F}_{\textrm{p}}$ and elastic deformation $\varvec{F}_{\textrm{e}}$ as follows:

1 $$\begin{aligned} \varvec{F} = \varvec{F}_{\textrm{e}} \cdot \varvec{F}_{\textrm{p}}. \end{aligned}$$

The plastic deformation gradient tensor transforms the reference configuration in the initial state into a new reference configuration as follows:

2 $$\begin{aligned} \textrm{d}\varvec{X} = \varvec{F}_{\textrm{p}} \cdot \textrm{d}\varvec{X}_{0}, \end{aligned}$$

where $\textrm{d}\varvec{X}_{0}$ and $\textrm{d} \varvec{X}$ represent the infinitesimal vectors before and after the spontaneous plastic deformation, respectively.

The spontaneous plastic deformation tensor is defined as follows:

3 $$\begin{aligned} \varvec{F}_\text {p} = \theta _{\zeta }(\varvec{X}_{0}) \varvec{\zeta }\otimes \varvec{\zeta } + \theta _{\eta }(\varvec{X}_{0}) \varvec{\eta }\otimes \varvec{\eta } + \varvec{\xi }\otimes \varvec{\xi }, \end{aligned}$$

where $\theta _{\zeta }$ and $\varvec{\zeta }$ represent the degree of plastic deformation and the unit vector along the longitudinal direction, respectively, and $\theta _{\eta }$ and $\varvec{\eta }$ are those along the circumferential direction, and $\varvec{\xi }$ is the unit vector along the thickness direction.

### Hyperelastic deformation

The elastic deformation gradient tensor further transforms the new reference configuration as:

4 $$\begin{aligned} \textrm{d}\varvec{x} = \varvec{F}_{\textrm{e}} \cdot \textrm{d} \varvec{X}, \end{aligned}$$

where $\textrm{d}\varvec{x}$ is the infinitesimal vector in the deformed configuration. The right Cauchy–Green deformation tensor and the Green–Lagrange strain tensor are defined as:

5 $$\begin{aligned} \varvec{C}_{\textrm{e}} = \varvec{F}^{\textrm{T}}_{\textrm{e}} \cdot \varvec{F}_{\textrm{e}}, \end{aligned}$$

and

6 $$\begin{aligned} \varvec{E}_{\textrm{e}} = \frac{1}{2} \left( \varvec{C}_{\textrm{e}} - \varvec{I} \right) , \end{aligned}$$

respectively, where $\varvec{I}$ is the identity tensor.

Using the variational formulation, the governing equation is given as follows:

7 $$\begin{aligned} \delta W = \int _{V}\varvec{S}_\textrm{e}:\delta \varvec{E}_\textrm{e}\textrm{d}V - \int _{A}\varvec{t}_0\cdot \delta \varvec{u}\textrm{d}A = 0, \end{aligned}$$

where $\delta W$ is the variation of the total energy, $\varvec{S}_\textrm{e}$ is the second Piola–Kirchhoff stress tensor associated with the hyperelastic deformation, $\delta \varvec{E}_{\textrm{e}}$ is the variation of the Green–Lagrange strain tensor, $\varvec{t}_{0}$ is the traction vector per unit area, $\delta \varvec{u}$ is the virtual displacement, and *V* and *A* are the volume and surface of the tube in the reference configuration after the plastic deformation. The second Piola–Kirchhoff stress tensor is obtained as follows:

8 $$\begin{aligned} \varvec{S}_{\textrm{e}} = \frac{\partial w}{\partial \varvec{E}_{\textrm{e}}} = 2 \frac{\partial w}{\partial \varvec{C}_{\textrm{e}}}, \end{aligned}$$

where *w* is the strain energy density function. We use the compressible neo-Hookean model (Bonet and Wood 2008):

9 $$\begin{aligned} w = \frac{\mu }{2}(I_{1} - 3) - \mu \ln J + \frac{\lambda }{2}\left( \ln J \right) ^{2}, \end{aligned}$$

where $\mu$ and $\lambda$ are the Lamé’s constants, $I_{1}$ is the first invariant of the right Cauchy–Green tensor, and $J = \textrm{det} \varvec{F}_{\textrm{e}}$. Lamé’s constants are obtained using Young’s modulus *E* and Poisson’s ratio $\nu$ as follows:

10 $$\begin{aligned} \mu&= \frac{E}{2(1+\nu )}, \end{aligned}$$

11 $$\begin{aligned} \lambda&= \frac{E\nu }{(1+\nu )(1-2\nu )}. \end{aligned}$$

In this study, we set the Poisson’s ratio to $\nu = 0.495$.

### Numerical methods

Using directional derivatives, the variational formulation is linearized for $\Delta \varvec{u}$ as follows:

12 $$\begin{aligned} \delta W(\varvec{u},\delta \varvec{u}) + {\mathcal {D}}\delta W(\varvec{u}, \delta \varvec{u})[\Delta \varvec{u}] =0, \end{aligned}$$

where ${\mathcal {D}}\delta W(\varvec{u}, \delta \varvec{u})[\Delta \varvec{u}]$ denotes the directional derivative of $\delta W$ at $\varvec{u}$ in the direction of $\Delta \varvec{u}$. We use the Newton–Raphson method to solve Eq. (7). For the spatial discretization, the isogeometric analysis (Borden et al. 2010; Hughes et al. 2005) is used, where the polynomial degree of the basis function is $p = 3$. The displacement $\varvec{u}$ is interpolated with the B-spline basis function *N* as

13 $$\begin{aligned} \varvec{u} = N^{\alpha } \varvec{u}_{\textrm{cp}}^{\alpha }, \end{aligned}$$

where $\varvec{u}_{\textrm{cp}}$ denotes the displacement vector of the control point. The discretized equation at the *k*-th iteration of the Newton–Raphson method is expressed as:

14 $$\begin{aligned} \varvec{K}^{k-1} \cdot \varvec{\Delta u}_{\textrm{cp}}^{k} = - \varvec{R}^{k-1}, \end{aligned}$$

where $\varvec{K}$ denotes the tangent stiffness matrix, $\varvec{\Delta u}_{\textrm{cp}}$ denotes the increment of the displacement vector of the control points, and $\varvec{R}$ denotes the equivalent nodal force vector.

We use the Newmark-$\beta$ method to stably compute large deformations, including buckling. In the Newmark-$\beta$ method, Eq. (14) is rewritten as:

15 $$\begin{aligned} \varvec{C} \cdot \dot{\varvec{u}}_{\tau +\Delta \tau }^{k} + \varvec{K}^{k-1}_{\tau +\Delta \tau } \cdot \varvec{\Delta u}_{\textrm{cp}}^{k} =-\varvec{R}^{k-1}_{\tau +\Delta \tau }, \end{aligned}$$

where $\tau$ is the pseudo time and $\varvec{C}$ is the damping matrix. The damping matrix of *e*-th element is given as follows:

16 $$\begin{aligned} \varvec{C}_{(e)} = \int _{V_{(e)}} \eta N^\alpha N^\beta \textrm{d}V, \end{aligned}$$

where $\eta$ is the damping coefficient. The displacement and velocity after $\Delta \tau$ are given by:

17 $$\begin{aligned} \varvec{u}_{\tau +\Delta \tau }&= \varvec{u}_{\tau } + \Delta \tau \dot{\varvec{u}}_{\tau } \nonumber \\&\quad + \Delta \tau ^2 \left\{ \left( \frac{1}{2} - \beta \right) \ddot{\varvec{u}}_{\tau } + \beta \ddot{\varvec{u}}_{\tau +\Delta \tau } \right\} , \end{aligned}$$

and

18 $$\begin{aligned} \dot{\varvec{u}}_{\tau +\Delta \tau } = \dot{\varvec{u}}_{\tau } + \Delta \tau \left\{ \gamma \ddot{\varvec{u}}_{\tau +\Delta \tau } + (1-\gamma ) \ddot{\varvec{u}}_\tau \right\} . \end{aligned}$$

We set $\gamma$ to 0.5 and $\beta$ to 0.25. The dimensionless damping coefficient of the Newmark-$\beta$ method is set at $\frac{\eta D}{E \Delta \tau } = 100$ (see Appendix A).

### Simulation conditions

Circumferential contraction and longitudinal relaxation are applied to the tube wall between $z = 0.5D$ and $z = 1.0D$ as shown in Fig. 1. The contraction and relaxation ratios are determined by sinusoidal distributions such that after the plastic deformation, the tube length is $L_{\textrm{max}}$ and the tube diameter at the most contracting region is $D_{\textrm{min}}$. Note that *L* and $L_{\textrm{max}}$ represent the reference tube lengths before and after plastic deformation, respectively. Both ends of the tube are fixed.

**Fig. 2:**
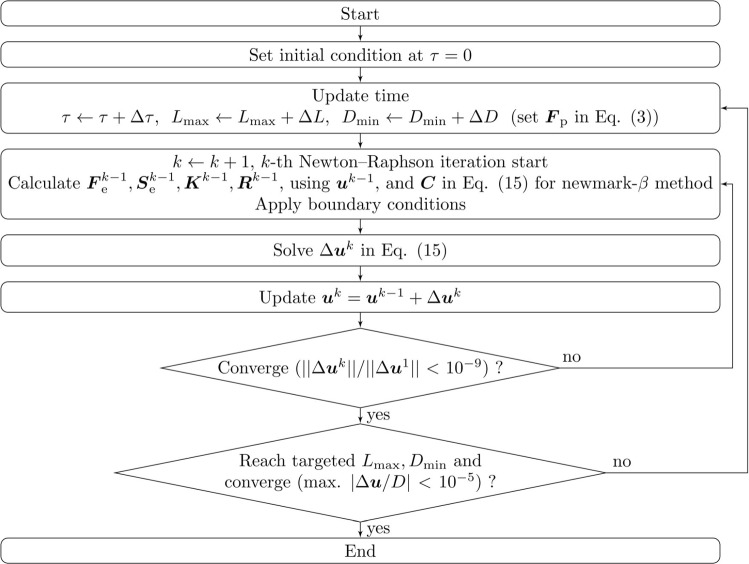
Flow chart of simulation

We developed an in-house solver for the isogeometric analysis. A flow chart of the simulation is shown in Fig. 2. At each pseudo time step, the tube length increases (decreases) by $\Delta L$, and the tube diameter decreases (increases) by $\Delta D$. This plastic deformation generates residual stresses that induce hyperelastic deformation. The hyperelastic deformation problem is solved by the Newton–Raphson method with the newmark-$\beta$ method.

We performed a mesh convergence test (Appendix A) and determined the number of control points in the longitudinal, circumferential, and thickness directions as $N_{\textrm{L}} = 305$, $N_{\textrm{C}} = 60$, and $N_{\textrm{H}} = 8$, respectively, where the mesh interval in the longitudinal direction is smaller for the contraction and relaxation region ($0.5D< z < 1.0D$) than other regions. The Newmark-$\beta$ method is iterated until the effect of the damping is ignored, and Eq. (15) converges to Eq. (14). The final deformed shapes are obtained when the maximum value of $|\Delta u_{\textrm{cp}}/D| < 10^{-5}$. Model parameters are summarized in Table 1.

**Table 1:** Model parameters

Symbol	Value	Description
*H*/*D*	0.06	Normalized thickness
*L*/*D*	3	Normalized length before plastic deformation
$D_{\textrm{min}}/D$	0.25	Ratio of diameters before and after plastic deformation
$L_{\textrm{max}}/L$	$1.0-1.25$	Ratio of lengths before and after plastic deformation
$\nu$	0.495	Poisson’s ratio
$\gamma$	0.5	Parameter for Newmark-$\beta$
$\beta$	0.25	Parameter for Newmark-$\beta$
$\eta D / E\Delta \tau$	100	Dimensionless damping coefficient
$N_\textrm{L}$	305	Number of control points in the longitudinal direction
$N_\textrm{C}$	60	Number of control points in the circumferential direction
$N_\textrm{H}$	8	Number of control points in the thickness direction

## Results

### Circumferential contraction with longitudinal relaxation causes invagination

**Fig. 3:**
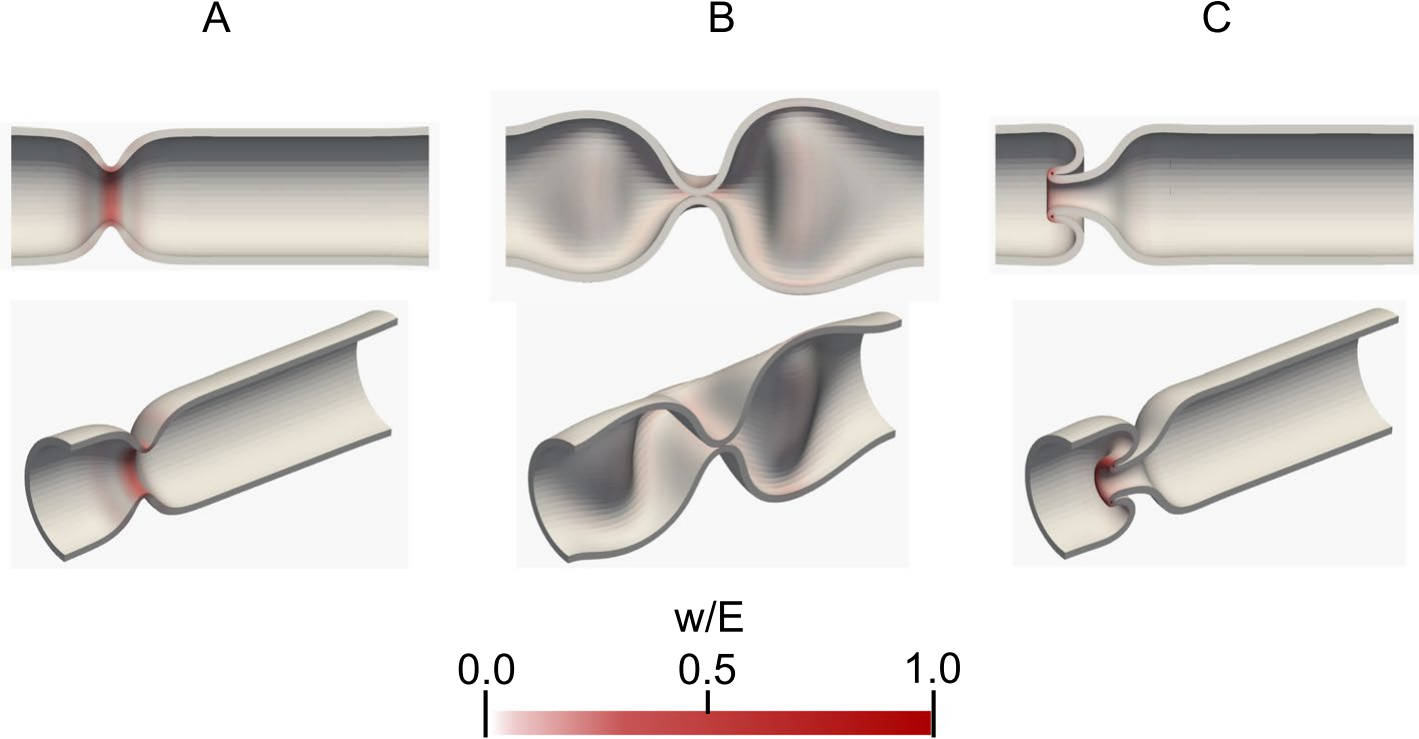
Typical examples of tube deformation. **A** V-shaped contraction by circumferential contraction $(L_{\textrm{max}}/L, D_{\textrm{min}}/D ) = (1.0, 0.25)$, **B** tube collapse by longitudinal relaxation $(L_{\textrm{max}}/L, D_{\textrm{min}}/D ) = (1.2, 1.0)$, and **C** tube invagination caused by circumferential contraction with longitudinal relaxation $(L_{\textrm{max}}/L, D_{\textrm{min}}/D ) = (1.25, 0.25)$. The contraction and relaxation are applied to $0.5 \le z/D \le 1.0$. The color shows the local strain energy

First, we examine tube deformation caused by circumferential contraction and longitudinal relaxation. As shown in Fig. 3A and B, neither the circumferential contraction nor the longitudinal relaxation causes invagination. A V-shaped contraction is formed by the circumferential contraction (Fig. 3A), whereas tube collapse occurs by longitudinal relaxation (Fig. 3B). Next, circumferential contraction and longitudinal relaxation are simultaneously applied to the tubes. Tube invagination is caused by circumferential contractions with longitudinal relaxation, as shown in Fig. 3C. Note that the tube invagination also occurs, even with perturbation in the boundary condition (see Appendix B).

**Fig. 4:**
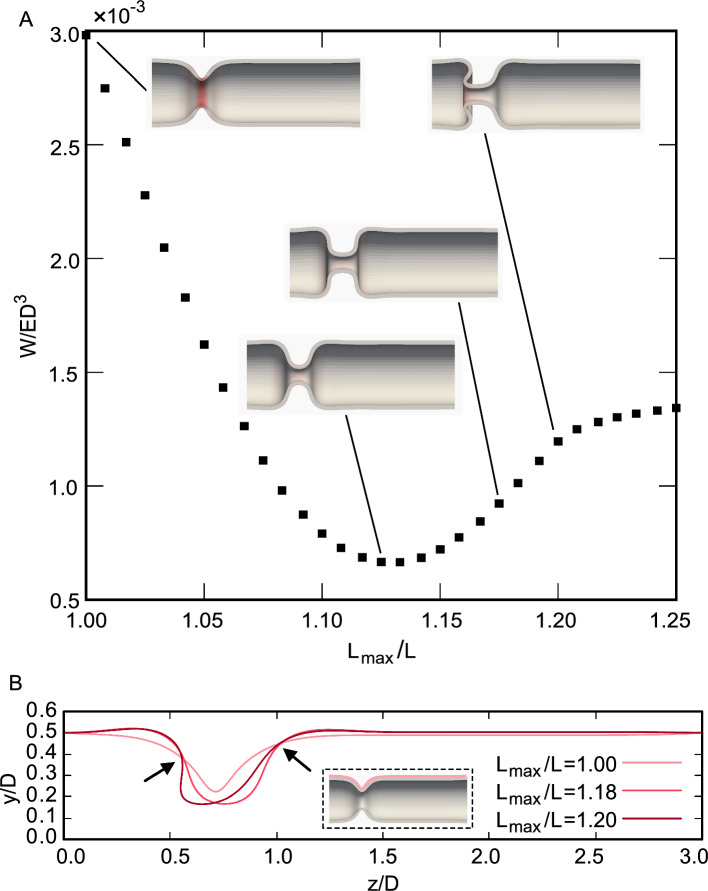
Effect of $L_{\textrm{max}}/L$ on the tube shape and strain energy. $D_{\textrm{min}}/D =$ 0.25. **A** Total strain energy. Inset figures show tube shapes. **B** Outer wall shapes at $L_{\textrm{max}}/L =$ 1.0, 1.18 and 1.2. Arrows show the centers of rotation

Figure 4A shows how the deformed shape and total strain energy change with $L_{\textrm{max}}/L$, where $D_{\textrm{min}}/D$ is fixed at 0.25 (see also Supplementary file). When longitudinal relaxation is applied, the shape of the contracting region changes from a V- to a U-shape. The total strain energy decreases with $L_{\textrm{max}}/L$ and reaches bottom peak at $L_{\textrm{max}}/L \sim 1.13$. This is because the tension generated by the circumferential contraction is released by the longitudinal relaxation. When $L_{\textrm{max}}/L$ increases further, the total strain energy increases, owing to an increase in the curvature in the contracting region. The contracting region then changes from a U-shape to a square shape and finally invaginates. After invagination, the gradient of the total strain energy decreases.

Figure 4B shows the shape of the outer wall of the tube. When $L_{\textrm{max}}/L$ increases, the wall of the contracting region rotates, resulting in a change in shape from a V-shape to invagination. In this case, the tube invaginates toward the side of the colon. The length of the colon side is shorter than that of the ileum, and the colon side is more compressed by longitudinal relaxation. Tube invagination may occur toward the more compressed side of the tube.

### Invagination occurs when tube forms square-shaped contraction

**Fig. 5:**
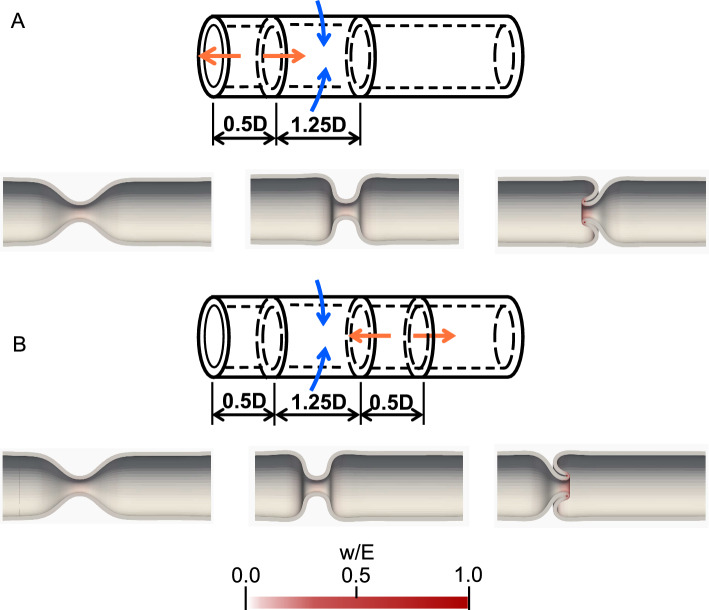
Tube deformation by longitudinal relaxation at **A** the colon side ($0.0 \le z/D \le 0.5$) and **B** the ileum side ($1.75 \le z/D \le 2.25$). The region of circumferential contraction is fixed to $0.5 \le z/D \le 1.75$. (left) $L_{\textrm{max}}/L = 1.0$, (center) $L_{\textrm{max}}/L = 1.17$, and (right) $L_{\textrm{max}}/L = 1.25$. $D_{\textrm{min}}/D = 0.25$

**Fig. 6:**
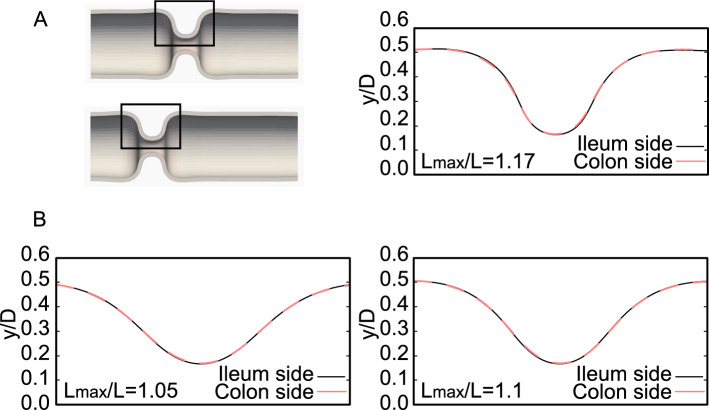
Outer wall shapes of contracting regions **A** at $L_{\textrm{max}}/L =$1.17, **B** $L_{\textrm{max}}/L =$1.05 and $L_{\textrm{max}}/L =$1.1. The outer wall shapes are nearly the same between the colon- and ileum-side relaxations

Circumferential contraction and longitudinal relaxation were applied to the same region of the tube in Sec. 3.1. To investigate the effect of the longitudinal relaxation position, we compare the tube deformation caused by the colon-side relaxation (Fig. 5A) with that by the ileum-side relaxation (Fig. 5B). In the case of the colon-side relaxation, when $L_{\textrm{max}}/L$ increases, the contracting region deforms into a square shape at $L_{\textrm{max}}/L \sim 1.17$ and then invaginates toward the colon side (Fig. 5A). In contrast, in the case of ileum-side relaxation, the contracting region also deforms into a square shape at $L_{\textrm{max}}/L \sim 1.17$ but invaginates toward the ileum (Fig. 5B). We find that before invagination, the shapes of the contracting regions are nearly the same, even though the relaxation positions are different (Fig. 6). These results suggest that the shape of the contracting region is determined by $L_{\textrm{max}}/L$ and $D_{\textrm{min}}/D$, and invagination occurs when the tube forms a square-shaped contracting region.

**Fig. 7:**
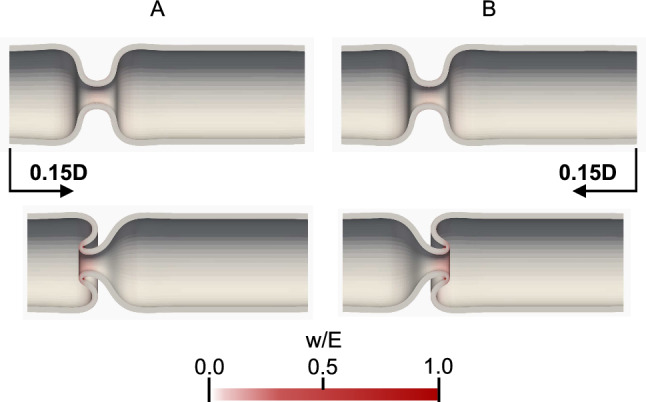
Tube deformation by forced displacement to **A** the colon-side end and **B** the ileum-side end. A forced displacement in the longitudinal direction is given to the deformed tube by the ileum-side relaxation at $L_{\textrm{max}}/L = 1.17$ (Fig. 5B, center)

Longitudinal compression to a square-shaped contracting region may induce invagination toward the compressed side, regardless of how the square-shaped contracting region is created. To verify this, we further examine tube deformation by forced displacements. For the deformed tube by ileum-side relaxation at $L_{\textrm{max}}/L = 1.17$ (Fig. 5B, center), a forced displacement (0.15*D*) is applied in the longitudinal direction to the colon-side end or the ileum-side end. When forced displacement is applied to the colon side, the tube invaginates toward the colon side (Fig. 7A), whereas when forced displacement is applied to the ileum side, the tube invaginates toward the ileum side (Fig. 7B).

## Discussion

The mechanism of intussusception has been explored in terms of its epidemiology and pathology; however, few studies have considered this mechanism from a mechanical viewpoint. We investigated the invagination of a hyperelastic tube using the isogeometric analysis.

We showed that the circumferential contraction with longitudinal relaxation of a hyperelastic tube causes invagination at the contracting region of the tube. Circumferential contraction corresponds to contraction of the circular muscle, and longitudinal relaxation corresponds to relaxation of the longitudinal muscle. In healthy subjects, circular and longitudinal muscles synchronize during peristalsis. The contraction of the longitudinal muscle is followed by that of the circular muscle (Korsapati et al. 2008; Nicosia et al. 2001; Pouderoux et al. 1997; Hur et al. 2021), and the peaks of the two muscle contractions almost coincide (Korsapati et al. 2008). Our results indicate that the uncoordinated motion of the circular and longitudinal muscles plays a significant role in the process of invagination.

The uncoordinated motion of these two muscles during peristalsis may be caused by inflammation. Pediatric intussusception is associated with the inflammation caused by bacterial or viral infections (Bines et al. 2006; Handa et al. 2021; Nylund et al. 2010). Previous studies have shown that administering lipopolysaccharide (LPS), an outer membrane component of gram-negative bacteria, induced both intussusception and inflammation in mice (Lin et al. 1998; Nissan et al. 1997; Sönmez et al. 2008). Reardon et al. (2013) reported that choline acetyltransferase (ChAT) was expressed in immune cells stimulated with LPS. Korsapati et al. (2008) demonstrated that acetylcholinesterase inhibitors can relax the longitudinal muscles while contracting the circular muscles. In addition, Nakamori et al. (2024) reported that administration of LPS changed the frequency of peristalsis in rats. Taken together, acetylcholine expression attributed to stimulation by LPS may induce uncoordinated motions of the circular and longitudinal muscles, leading to invagination of the intestinal wall.

In clinical practice, ultrasonography or abdominal computed tomography are used to confirm an intussusception (Byrne et al. 2005). However, predicting the risk of intussusception remains challenging using medical images before the onset of intussusception or after treatment. We found that invagination occurred when a square-shaped contracting region appeared. This result suggests that the configuration of peristalsis may serve as an indicator of the risk of intussusception.

The incidence of intussusception is low. For example, the annual incidence of hospital admissions due to intussusception ranges from 34 to 90 per 100,000 children under 1 year of age (Clark et al. 2019). In this study, we did not consider the recovery from invagination. Peristalsis is a periodic wall motion in which the circular muscles relax after contraction, and the longitudinal muscles contract after relaxation. The invagination of the intestine may occur more often than is actually diagnosed. However, it is possible that a significant proportion of these cases recover during peristalsis, and only a small number of cases progress to intussusception. This possibility was also suggested in a previous study that used mice as subjects (Lin et al. 1998).

Using a simple continuum mechanics model, we have demonstrated that tube invagination occurs as a mechanical buckling problem. In reality, the intestinal shape is more complex, the intestinal wall has a layered structure, and the intestinal wall motion results from chemical, electrical, and mechanical coupling. Therefore, our findings need to be confirmed in actual clinical cases. For example, the presence of a lead point has been suggested in ileocolic intussusception. Thus, it is important to identify the mechanical role of the lead point in pediatric intussusception. Further studies are needed to deepen our understanding of intestinal invagination. However, we hope that this study will contribute to advancing knowledge in this field and assist in the prediction and diagnosis of intussusception in the future.

## Supplementary Information

Below is the link to the electronic supplementary material.

Supplementary file 1 (mp4 347 KB)

## Figures and Tables

**Fig. 8:**
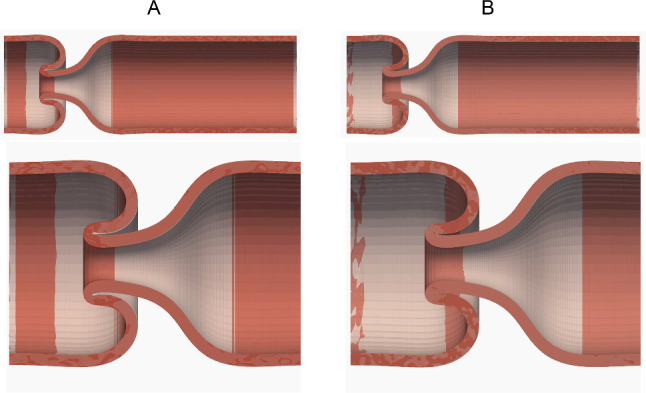
Convergence test. **A** Overlapped cross-sectional views for $(N_{\textrm{L}}, N_{\textrm{C}}, N_{\textrm{H}}) = (305, 60, 8)$ (white) and $(N_{\textrm{L}}, N_{\textrm{C}}, N_{\textrm{H}}) = (201, 60, 8)$ (red). **B** Overlapped cross-sectional views for $\frac{\eta D}{E \Delta \tau } = 100$ (white) and $\frac{\eta D}{E \Delta \tau } = 10$ (red)

**Fig. 9:**
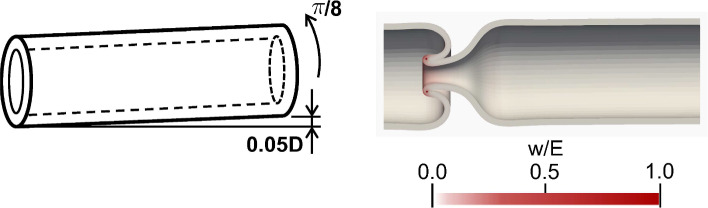
Tube invagination at $(L_{\textrm{max}}/L, D_{\textrm{min}}/D) = (1.25,0.25)$ with a displacement of 0.05*D* in the *y*-direction, and a rotation of $\pi /8$ on the ileum-side end

## Data Availability

No datasets were generated or analyzed during the current study.

## Appendix A: Convergence test

A mesh convergence test was conducted. Figure 8A shows the tube shapes between $(N_{\textrm{L}}, N_{\textrm{C}}, N_{\textrm{H}}) = (201, 60, 8)$ and $(N_{\textrm{L}}, N_{\textrm{C}}, N_{\textrm{H}}) = (305, 60, 8)$. The tube shapes are in good agreement; thus, we decided to use $(N_{\textrm{L}}, N_{\textrm{C}}, N_{\textrm{H}}) = (305, 60, 8)$.

We also examined the effect of the damping coefficient using the Newmark-$\beta$ method. Figure 8B shows a comparison between $\frac{\eta D}{E \Delta \tau } = 100$ and $\frac{\eta D}{E \Delta \tau } = 10$. Therefore, we decided to use $\frac{\eta D}{E \Delta \tau } = 100$.

## Appendix B: Effect of boundary condition

We tested the effect of the boundary conditions on tube invagination. A displacement of 0.05*D* and rotation of $\pi /8$ were applied to the ileum-side end. The invagination of the tube occurred, as shown in Fig. 9.
